# The Effect of Platelet-Rich Plasma on Healing Time in Patients Following Pilonidal Sinus Surgery: A Systematic Review

**DOI:** 10.7759/cureus.27777

**Published:** 2022-08-08

**Authors:** Qaisar I Khan, Hassan Baig, Abdulaziz Al Failakawi, Saad Majeed, Mujahid Khan, James Lucocq

**Affiliations:** 1 Department of General Surgery, Queen Elizabeth University Hospital, Glasgow, GBR; 2 Department of General Surgery, Sabah Hospital, Kuwait, KWT; 3 Department of Medical Education, University of Aberdeen, Aberdeen, GBR; 4 Department of Emergency Medicine, University Hospital Ayr, Ayr, GBR; 5 School of Medicine, University of Glasgow, Glasgow, GBR; 6 Department of General Surgery, Victoria Hospital, Kirkcaldy, GBR

**Keywords:** a systematic review, return to work, healing time, pilonidal sinus surgery, platelet-rich plasma/ prp

## Abstract

Background: Pilonidal disease (PD) is a debilitating condition characterised by the infection of subcutaneous tissue in the sacrococcygeal area. It is associated with a high risk of recurrence, pain, infection, and purulent discharge. The two main surgical methods of pilonidal sinus disease include excision with primary closure/flap repair or excision of the sinus with healing by secondary intent. Wounds left open to heal by secondary intent remain extremely common due to their association with reduced risk of recurrence, however, it is associated with prolonged healing times. This study aims to determine whether platelet-rich plasma (PRP) reduces healing time in patients post pilonidal sinus surgery with healing by secondary intent compared to simple wound dressings.

Method: Six databases were searched from their date of origin to May 30, 2022 for randomised control trials using predetermined inclusion and exclusion criteria. Only four papers were selected for review as per the Population, Intervention, Comparison, Outcomes and Study design (PICOS) criteria. Critical appraisal was carried out according to the Scottish Intercollegiate Guidelines Network Methodology Checklist for Randomised Control Trials and was assessed for risk of bias according to the Cochrane Handbook for Systematic Review of Interventions. The pooled effect size was calculated using the fixed-effect model. A homogeneity of pooled effect size for the studies was also found (Cochrane Q test, p-value = 0.97 I-square = 0.0%).

Result: Four studies (n = 336) were included in this review. Three of the four studies reported a statistically significant reduction in time taken in healing the wound. The mean difference between the intervention (PRP group) and the control group was 13.01 days, (95% CI 12.15-13.86 days, p < 0.00001). All of the included studies also reported a statistically significant reduction in time taken to return to work/activities of daily living in the treatment group compared to the control group (MD 9.68 days, 95% CI 9.16-10.21 days, p < 0.00001).

Conclusion: This study shows that PRP is effective in reducing healing time and is associated with a significantly shorter period taken to return to work/activities of daily living in patients post pilonidal sinus surgery, which was the primary and secondary outcome investigated in this systematic review, respectively. PRP should routinely be offered to patients undergoing excisional pilonidal sinus surgery for the aforementioned benefits.

## Introduction and background

Pilonidal disease (PD) is a very debilitating condition and is diagnosed as the infection of the skin and subcutaneous tissue in the midline of the natal cleft in the sacrococcygeal area and has been reported in the literature since 1883 [1,2]. The term ‘pilonidal’ is derived from Latin; ‘pilus’ meaning hair and ‘nidus’ meaning nest [3]. It can occur at any age but most commonly affects young male patients aged 15-40 years. It has an incidence of approximately 26 per 100,000 people [4]. Its presentation varies from asymptomatic pits to symptomatic with pain, chronic discharge, and low quality of life [5].

Initially, PD was considered congenital in origin, but recent studies report that it is an acquired disease. Although the pathogenesis of PD is not known, it is thought that it occurs due to trapped hair follicles in the skin of the natal cleft [6]. The presence of hair follicles causes foreign body reactions and foreign body granuloma [7]. Although the exact aetiology is unknown, the risk factors include male sex, hirsute body habitus, and family history [4]. Some of the complications of PD include recurrence, delayed wound healing, abscess, and in rare cases, osteomyelitis, or malignancy [8].

The diagnosis of PD is clinical and does not require any blood tests or diagnostic imaging. Management for PD can be non-surgical and/or surgical. Non-surgical management includes cryosurgery, regular shaving, and phenol application. Surgical management includes excision with primary closure/flap repair or open excision of the sinus followed by healing by secondary intent. Excision of the pilonidal sinus followed by flap repair is generally considered to have low recurrence rates, as is excision of the pilonidal sinus with healing by secondary intent, although this is associated with significantly increased healing time. Multiple flap techniques are used in current practice such as rotational flaps, and Karydakis and Limberg flaps to manage pilonidal sinus disease [9]. The other common method is excision of the pilonidal sinus followed by healing by secondary intent. In this method, after excision of a wide-field surface, the wound is laid open and allowed to heal by granulation. This method results in a longer healing time and requires daily dressing of the wound [10]. Therefore, one of the key issues with this commonly practiced method remains the speed of recovery.

The healing process of a wound includes three stages: inflammation, tissue formation, and tissue remodelling. This process involves the recruitment of neutrophils and transformation of monocytes which leads to phagocytosis and degrading of necrotic tissue. This causes the production of numerous growth factors such as vascular endothelial growth factors (VEGF), transforming growth factor beta (TGF-β), and platelet-derived growth factor (PDGF) which promote cell proliferation and angiogenesis [11].

Numerous studies have reported the administration of platelet-rich plasma (PRP) to the wound to promote the healing process. It has been used to speed up healing in maxillofacial, plastic surgeries, and venous and diabetic foot ulcers [12-14]. PRP contains growth factors and chemokines such as VEGF, TGF-β, and PDGF which play a key role in proliferation and angiogenesis [15]. There are various methods in use for the extraction of PRP. The most common method is by first obtaining autologous blood, followed by centrifuging the blood to obtain the PRP, which is applied to the wound either in a form of gel, solution, or injection.

The primary objective of this study was to investigate the effect of PRP in patients undergoing excision of the sinus followed by healing by secondary intent since healing time remains the major morbidity associated with this particular method. The secondary objectives aimed to explore the reduction in time taken to return to work/activities of daily living and the effect of PRP application in reducing post-operative wound infection.

## Review

Materials and methods

Inclusion and exclusion criteria were generated using Population, Intervention, Comparison, Outcomes and Study design (PICOS) model, summarised in Table 1.

**Table 1:** Literature search inclusion and exclusion criteria

	Inclusion	Exclusion
Patient	Adults > 16 years old, post-pilonidal surgery	Anyone < 16 years old, patients who didn’t undergo pilonidal surgery
Intervention	Pilonidal sinus surgery healing by secondary intent and application of platelet-rich plasma (PRP) post-surgery	Pilonidal sinus surgery with primary closure, no platelet-rich plasma or any other treatment modalities
Control	Pilonidal sinus surgery healing by secondary intent with simple wound dressing	Pilonidal sinus surgery with primary closure, control group using any other interventions
Outcome	Time taken to heal and/or time taken to return to work/activites of daily living	Studies not including time taken to heal
Study	Randomised control trials, total number of participants > 50, articles published in English	Trials which are not randomised control trials, total number of participants < 50, articles published in languages other than English

Randomised control trials (RCTs) comparing PRP with simple wound dressings in patients post-PD surgery with an open healing method were reviewed in this study. This study included adult participants over the age of 16 undergoing pilonidal sinus surgery with healing through secondary intent. The included randomised control trials compared PRP with either placebo or with simple wound dressings.

Objectives

The primary objective of this study was to investigate the effect of PRP on healing by secondary intent of pilonidal sinus wounds, postoperatively. Secondary objectives aimed to explore the effect of PRP application on reduction in time taken to return to work/activities of daily living and post-operative wound infection.

Search methods for identification of studies 

The following databases were searched to obtain relevant RCTs:

a. MEDLINE Ovid

b. EMBASE Ovid

c. Web of Science Ovid

d. Scopus

e. Cochrane Central Register of Controlled Trials

f. Clinicaltrials.gov

**Figure 1:**
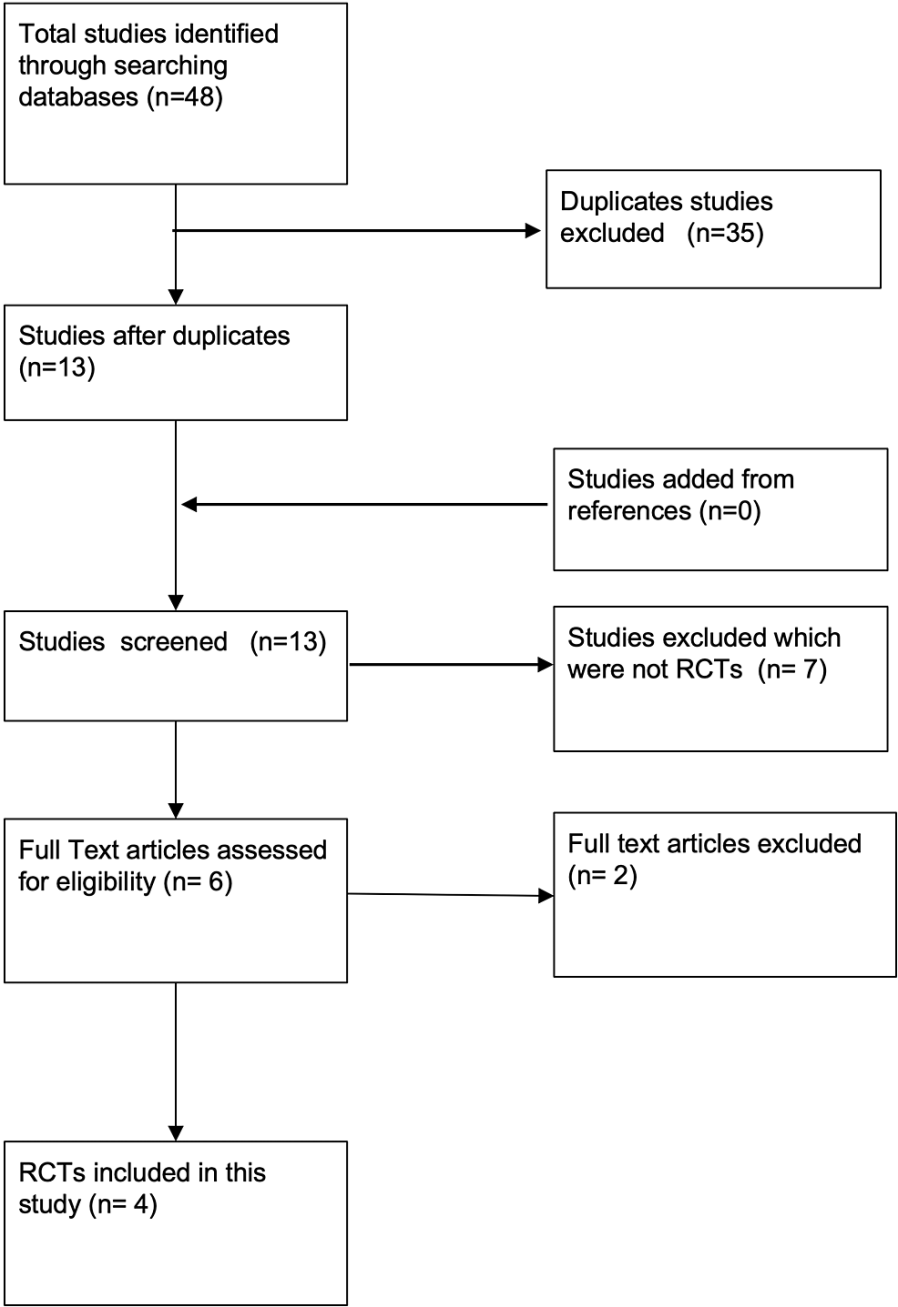
PRISMA flow diagram of the literature search results PRISMA: Preferred Reporting Items for Systematic Reviews and Meta-Analyses

These databases were searched from their date of origin until May 30, 2022, to obtain as many RCTs as possible, and to minimise any bias. In total, 48 research papers were obtained through this process. After the removal of duplicates, the remaining papers were analysed by title, followed by abstract, and lastly by their full text. In addition to this, the references of the relevant papers were cross-checked to identify any further studies. Restrictions were placed with respect to the language (English), however, no restrictions were placed with respect to the date of publication. At the end of this literature search process, four RCTs [16-19] fulfilled the PICOS criteria and were selected for review in this study. This process was carried out using Preferred Reporting Items for Systematic Reviews and Meta-Analyses (PRISMA) guidelines and is summarised in Figure 1.

All identified abstracts from the search strategies were assessed. Studies that were deemed irrelevant were excluded. Full-text publications were obtained for the abstracts to be formally assessed against the PICOS criteria. The studies were then analysed through a blind review process with the author and journal names remaining hidden.

The data extracted from the included studies were recorded. The data from the studies that were published in duplicate were recorded once. The following data were gathered from the studies:

a. Study information (first author, year of publication, location of care and country)

b. Method of study (study design, randomisation process)

c. Study population data (age, gender, total participants, and participants in each study arm)

d. Intervention (PRP)

e. Follow-up duration

f. Primary outcome (time to heal in days)

g. Secondary outcomes (time to return to work in days, wound infection)

The characteristics of the included studies are summarised in Table 2.

**Table 2:** Characteristics of the included studies M = Male; F = Female

Study	Study location and duration	Population	Control	Intervention	Mean age (Years)		Sample Size			Follow-up Duration
					Control	Intervention	Control	Intervention	Total	
Bahar et al. 2013 [16]	Imam Reza hospital, Iran 2011	All patients referred to surgical unit with acute pilonidal abscess	Extensive surgical removal and dressing	Extensive surgical removal and application of PRP PRP injected 24-36 hours after surgery	24.7 + 1.50	24.81 + 3.89	37 (M = 22, F = 15 )	37 (M = 20, F = 17)	74 (M = 42, F = 32)	Followed up until complete healing of the wound.
Gohar et al. 2020 [17]	Kafr El-Sheik hospital, Egypt Dec 2018 – Dec 2019	All patients with pilonidal sinus who underwent Lay Open excision technique	Total excision of pilonidal sinus using lay-open technique and dressing	Total excision of pilonidal sinus using lay-open technique and PRP injection on day 4 and 12	26.27± 4.62	25.07 ± 4.83	50 (M = 43 male, F = 7)	50 (M = 40, F = 10)	100. (M = 83, F = 17)	Followed up until complete healing of the wound.
Mohammadi et al. 2016 [18]	Shariati hospital, Iran June 2012 – Sep 2015	Patients with pilonidal sinus with a scheduled surgery	Wide excision and classic dressing with absorbent sterile cotton gauze	Wide excision and PRP injection right after surgery and continued weekly	27.49 ± 4.8	29.83 ± 7.04	55 (M = 52, F = 3)	55 (M = 54, F = 1)	110. (M = 106, F = 4)	Followed up until complete healing of the wound.
Spyridakis et al. 2009 [19]	University Hospital of Larissa, Greece 2006-2007	Patients with sacrococcygeal pilonidal disease	Total excision of the sinus and the wound remained open for secondary healing and dressing	Total excision of the sinus with secondary healing intentions and PRP gel applied on post-operative days 4 and 12	Not Available	Not Available	22 (M = 22, F = 0)	30 (M = 30, F = 0)	52 (M = 52, F = 0)	Followed up until post-operative day 30.

Critical appraisal

For this study, all papers were blindly analysed using Scottish Intercollegiate Guidelines Network (SIGN) Methodology Checklist for Randomised Control Trials [20] 

Summary of the search terms used and the summary of the SIGN checklist are presented in the appendix (Tables 3-4).

Risk of bias

**Figure 2:**
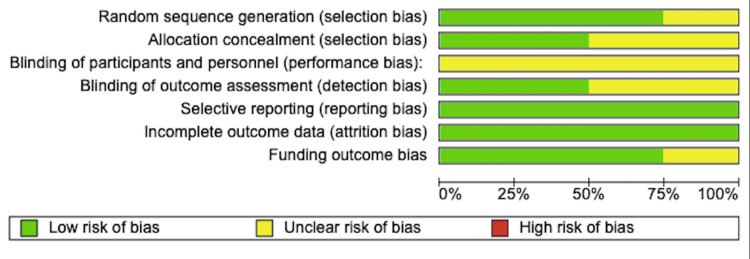
Risk of bias graph

The included studies were assessed for risk of bias using the Cochrane Handbook for Systematic Reviews of Interventions [21]. The papers that meet the criteria were classified as low risk, while failure to meet the criteria were classified as high risk of bias. Studies lacking sufficient details to be classified were classified as unclear risk. The domains assessed during the risk of bias assessment are summarised in Figure 2.

Random Sequence Generation (Selection Bias)

Three of the four studies provided sufficient details regarding randomisation allocation. Mohammadi et al. [18] used randomly permuted blocks, while Spyridakis et al. [19] and Gohar et al. [17] used sealed envelopes. Bahar et al. [16] did not give enough information hence, the risk of selection bias was classified as unclear.

Allocation Concealment (Selection Bias)

Two of the studies [17,19] used an adequate method of concealment using a sealed envelope. While in the study by Bahar et al. [16] and Mohammadi et al. [18] the risk of bias was unclear.

Blinding of Participants and Personnel (Performance Bias)

None of the studies gave sufficient details on whether both participants and personnel were blinded. Therefore, all of the RCTs [16-19] included in this study were considered as having ‘unclear risk’ for performance bias.

Blinding of Outcome Assessment (detection bias)

Bahar et al. [16] and Spyridakis et al. [19] were considered as having a low risk for detection bias as the surgeons who assessed the wound were blinded to the patient's treatment plan. While remaining two studies [17,18] was not clear whether the assessors were blinded.

Selective Reporting (Reporting Bias)

All four papers [16-19] reported the primary outcomes of this review and hence were considered as having a low risk of reporting bias.

Incomplete Outcome Data (Attrition Bias)

All four studies [16-19] reported complete data and hence had a low risk of attrition bias.

Funding Outcome Bias

Spyridakis et al. [19] had an unclear risk of funding outcome bias as the study did not declare any funding or conflict of interest. Mohammadi et al. [18] were funded by the university and were free from financial support from any commercial company. Therefore, it was considered as having a low risk for funding outcome bias. The remaining papers [16,17] declared no conflict of interest and thus were considered as having a low risk of funding outcome bias.

Results

Primary Outcome

**Figure 3:**
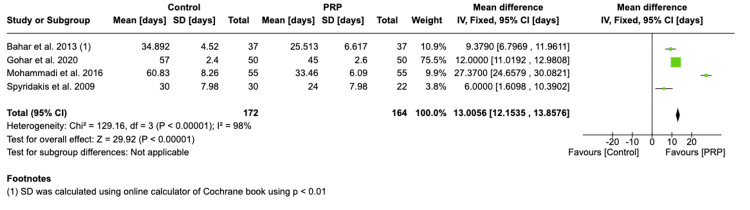
Forest plot of mean difference in time taken to heal between the platelet-rich plasma (PRP) group and control group Bahar et al. 2013 [16], Gohar et al. 2020 [17], Mohammadi et al. 2016 [18], Spyridakis et al. 2009 [19]

All studies [16-19] investigated the effect of PRP on healing time, although all four studies reported reduced healing time in the PRP group compared to the control group. Bahar et al. [16] showed no statistical significance. The mean time for wound healing was calculated as 32 days in the PRP group compared to 45.7 days in the control group. The analysis shows that the PRP group has a reduced healing time as compared with the control group (MD 13.01 days, 95% CI 12.15-13.86 days, p < 0.00001) (Figure 3).

**Figure 4:**
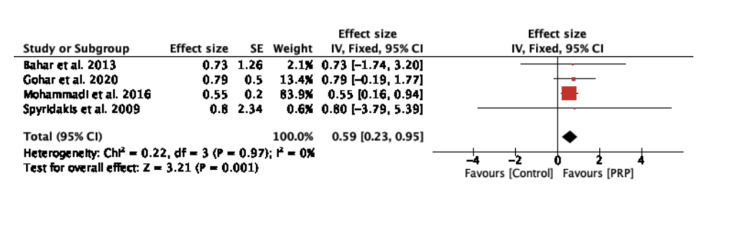
Forest plot of effect size in time to heal between the platelet-rich plasma (PRP) group and control group Bahar et al. 2013 [16], Gohar et al. 2020 [17], Mohammadi et al. 2016 [18], Spyridakis et al. 2009 [19]

The effect size was calculated as the ratio of post-operative wound healing time in the PRP group compared with the control group (Figure 4). The pooled effect size was 0.59 (95% CI: 0.23-0.95, p = 0.001) and was calculated using the fixed-effect model, suggesting that the healing time in the PRP group is 40% less than the healing time in the control group.

Secondary Outcomes

**Figure 5:**
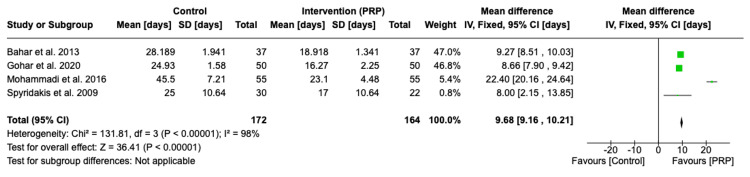
Forest plot of mean difference in time taken to return to work/activities of daily living between the platelet-rich plasma (PRP) group and control group Bahar et al. 2013 [16], Gohar et al. 2020 [17], Mohammadi et al. 2016 [18], Spyridakis et al. 2009 [19]

All of the included studies [16-19] reported a statistically significant reduction in time to return to work or activities of daily living in the PRP group compared to the control group (MD 9.68 days, 95% CI 9.16-10.21 days, p < 0.00001). The mean time to return to work or normal activities in the PRP group was 18.8 days compared to 30.9 days in the control group (Figure 5).

**Figure 6:**
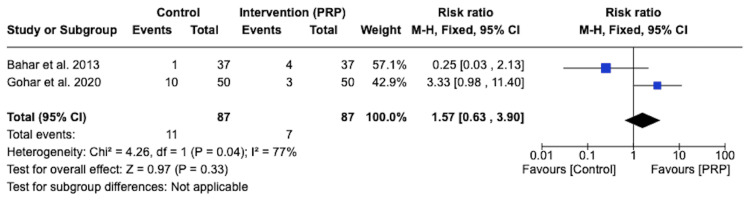
Forest plot of the risk ratio of infection rate in the platelet-rich plasma (PRP) group and control group Bahar et al. 2013 [16], Gohar et al. 2020 [17]

Only two of the studies [16,17] reported post-operative wound infection. There was no statistically significant difference between the PRP group and the control group in relation to the incidence of post-operative wound infection (RR1.57, 95% CI 0.63-3.90, p = 0.33) (Figure 6).

Discussion

Patients undergoing pilonidal sinus surgery with healing by secondary intent have a low recurrence rate. However, it is associated with a longer healing time, resulting in a delay in return to work or daily activities, pain, and reduced quality of life. Therefore, it is important to accelerate the wound healing process.

The primary objective of this study was to investigate the effect of PRP on healing by secondary intent following pilonidal sinus wounds, postoperatively. Wound healing in the PRP group was faster than in the control group. The mean difference between the PRP group and the control group was 13.01 days (95% CI 12.15-13.86 days, p < 0.00001). Additionallthe y, time taken to return to work/activities of daily living was much shorter in patients who received PRP (18.8 days) than in the patients in the control group (30.9 days).

Bahar et al. [16] also looked into the effect of PRP on pain severity measured via a pain linear visual scale. According to this study, the treatment group experienced significantly less pain than the control group (1.000 + 0.00 in PRP vs 1.973 + 0.164 in control, p = 0.000). They also reported a higher infection rate in the PRP group than in the control group. They proposed that it might be due to PRP providing optimal conditions for bacterial growth.

Spyridakis et al. [19] quantitatively reported the quality of life (including physical and mental health) in both groups. Patients who were treated with PRP scored significantly higher (75 points + 4.2, p < 0.03) compared to the control group (62 points + 5.6, p < 0.03).

In terms of the limitations of this review, none of the studies included in this systematic review provided sufficient details regarding the blinding process. Furthermore, there was no information as to whether the surgeries were performed by a single surgeon or varying surgeons. In addition to this, none of the included studies mentioned the exact volume of PRP used. Bahar et al. [16] and Mohammadi et al. [18] reported that the volume of PRP was dependent on the size of the pilonidal cavity but did not specify the exact volume.

The overall findings support the hypothesis that PRP causes a reduction in healing time and time taken to return to work/activities of daily living. However, there was no statistically significant benefit in the use of PRP to reduce post-operative wound infection. Therefore, these findings also support the hypothesis that the PRP group would have a shorter inpatient stay, thus posing a smaller economic burden on the healthcare system given the current associated costs of managing this disease. This is an important finding given this method of management remains commonplace in many healthcare systems despite management methods such as excision with flap repair being readily practiced.

## Conclusions

Overall, it can be concluded that this study favours the use of PRP compared to simple wound dressings in managing pilonidal sinus disease. This study concludes that the PRP therapy results in significantly reduced healing time as well as a shorter time taken to return to work or activities of daily living. The study also reveals that the efficacy of PRP therapy would likely reduce the economic burden associated with this condition. Given its effectiveness, PRP therapy should routinely be offered in conjunction with operative intervention in the management of PD for these reasons. We suggest that well-designed and multi-centre trials should be performed to look into the effect of PRP on other aspects of quality of life such as pain, infections, recurrence, and antibiotic consumption as well as its cost-effectiveness. Additionally, there is scope to further research the use of PRP in other surgical management options for pilonidal sinus disease patients such as excision with primary closure or flap repair, to further provide insight into the most efficient and optimum method for management.

## Appendices

**Table 3:** Summary of the search terms used

Search Method:	Search Terms:
MEDLINE Ovid	Pilonidal disease.mp Pilonidal sinus/ Pilonidal abscess.mp Sacrococcygeal region/ 1 or 2 or 3 or 4 Platelet rich plasm.mp Platelet-rich plasma/ PRP.mp 6 or 7 or 8 Randomised clinical trials.mp Randomized clinical trials.mp RCT.mp 10 or 11 or 12 5 and 9 and 13
EMBASE Ovid	Pilonidal disease.mp Pilonidal sinus/ Pilonidal abscess.mp 1 or 2 or 3 Platelet rich plasma.mp PRP.mp Thrombocyte rich plasma/ 5 or 6 or 7 Randomized controlled trials/ Randomi*ed controlled trials.mp RCT.mp 9 or 10 or 11 4 and 8 and 12
Web of Science Ovid	TOPIC: (Pilonidal disease) TOPIC: (Pilonidal sinus) #2 OR #1 TOPIC: (Platelet rich plasma) TOPIC: (PRP) ALL FIELDS: (Randomized control trial) #5 OR #4 #7 AND #3
Scopus	TITLE-ABS-KEY ( Pilonidal disease ) OR TITLE-ABS-KEY ( Pilonidal sinus) OR TITLE-ABS-KEY ( Pilonidal abscess ) AND TITLE-ABS-KEY (Platelet rich plasma) OR TITLE-ABS-KEY (PRP) (Filter: English Language, Article
Cochrane Central Register of Controlled Trials	MeSH descriptor: [Pilonidal disease] explode all trees MeSH descriptor: [Pilonidal sinus] explode all trees MeSH descriptor: [Pilonidal abscess] explode all trees (1 or 2 or 3) MeSH descriptor: [Platelet rich plasma] explode all trees MeSH descriptor: [PRP] explode all trees (5 or 6) (4 and 7)
Clinicaltrials.gov	1.Condition: Pilonidal disease 2.Other term: Platelet rich plasma Filters were used. Status: completed

**Table 4:** Summary of Scottish Intercollegiate Guidelines Network methodology checklist for randomised controlled trials Y = Yes N = No C = Cannot say N/A = Not Applicable Ia = Intervention arm Ca = Control arm ++ = High quality + = Acceptable - = Low quality

	Bahar et al. 2013 [16]	Gohar et al. 2020 [17]	Mohammadi et al. 2016 [18]	Spyridakis et al. 2009 [19]
The study addresses an appropriate and clearly focused question	Y	Y	Y	Y
The assignment of subjects to treatment groups is randomized	Y	Y	Y	Y
An adequate concealment method is used	C	Y	C	Y
The design keeps subjects and investigators ‘blind’ about treatment allocation	C	C	C	Y
The treatment and control groups are similar at the start of the trial.	N	Y	Y	Y
The only difference between groups is the treatment under investigation.	Y	Y	Y	Y
All relevant outcomes are measured in a standard, valid and reliable way.	Y	Y	Y	Y
What percentage of the individuals or clusters recruited into each treatment arm of the study dropped out before the study was completed?	I_a_ = 0% C_a_= 0%	I_a_ = 0% C_a_= 0%	I_a_ = 0% C_a_= 0%	I_a_ = 0% C_a_= 13.6%
All the subjects are analysed in the groups to which they were randomly allocated (often referred to as intention to treat analysis).	Y	Y	Y	Y
Where the study is carried out at more than one site, results are comparable for all sites.	N/A	N/A	N/A	N/A
How well was the study done to minimise bias?	-	+	+	++
Taking into account clinical considerations, your evaluation of the methodology used, and the statistical power of the study, are you certain that the overall effect is due to the study intervention?	Y	Y	Y	Y
Are the results of this study directly applicable to the patient group targeted by this guideline?	Y	Y	Y	Y

## References

[1] Farrell D, Murphy S (2011). Negative pressure wound therapy for recurrent pilonidal disease: a review of the literature. J Wound Ostomy Continence Nurs.

[2] Luedi MM, Schober P, Stauffer VK, Diekmann M, Doll D (2020). Global gender differences in pilonidal sinus disease: a random-effects meta-analysis. World J Surg.

[3] Kanat BH, Sözen S (2015). Disease that should be remembered: sacrococcygeal pilonidal sinus disease and short history. World J Clin Cases.

[4] Søndenaa K, Andersen E, Nesvik I, Søreide JA (1995). Patient characteristics and symptoms in chronic pilonidal sinus disease. Int J Colorectal Dis.

[5] Humphries AE, Duncan JE (2010). Evaluation and management of pilonidal disease. Surg Clin North Am.

[6] Hodges RM (1880). Pilo-nidal sinus. Boston Med Surg J.

[7] Patey DH, Scarff RW (1946). Pathology of the postanal pilonidal sinus; its bearing on treatment. Lancet.

[8] De Bree E, Zoetmulder FAN, Christodoulakis M Treatment of malignancy arising in pilonidal disease. Ann Surg Oncol.

[9] Editorial Editorial (2021). Pilonidal sinus disease: early surgery and the limberg flap improve patient outcomes. Adv Skin Wound Care.

[10] McCallum IJ, King PM, Bruce J (2008). Healing by primary closure versus open healing after surgery for pilonidal sinus: systematic review and meta-analysis. BMJ.

[11] Lindeboom JA, Mathura KR, Aartman IH, Kroon FH, Milstein DM, Ince C (2007). Influence of the application of platelet-enriched plasma in oral mucosal wound healing. Clin Oral Implants Res.

[12] Serra-Mestre JM, Serra-Renom JM, Martinez L, Almadori A, D'Andrea F (2014). Platelet-rich plasma mixed-fat grafting: a reasonable prosurvival strategy for fat grafts?. Aesthetic Plast Surg.

[13] Mohammadi MH, Molavi B, Mohammadi S (2017). Evaluation of wound healing in diabetic foot ulcer using platelet-rich plasma gel: a single-arm clinical trial. Transfus Apher Sci.

[14] Ferrara N, Henzel WJ (1989). Pituitary follicular cells secrete a novel heparin-binding growth factor specific for vascular endothelial cells. Biochem Biophys Res Commun.

[15] Marlovits S, Mousavi M, Gäbler C, Erdös J, Vécsei V (2004). A new simplified technique for producing platelet-rich plasma: a short technical note. Eur Spine J.

[16] Bahar MM, Akbarian MA, Azadmand A (2013). Investigating the effect of autologous platelet-rich plasma on pain in patients with pilonidal abscess treated with surgical removal of extensive tissue. Iran Red Cres Med J.

[17] Gohar MM, Ali RF, Ismail KA, Ismail TA, Nosair NA (2020). Assessment of the effect of platelet rich plasma on the healing of operated sacrococcygeal pilonidal sinus by lay-open technique: a randomized clinical trial. BMC Surg.

[18] Mohammadi S, Nasiri S, Mohammadi MH (2017). Evaluation of platelet-rich plasma gel potential in acceleration of wound healing duration in patients underwent pilonidal sinus surgery: a randomized controlled parallel clinical trial. Transfus Apher Sci.

[19] Spyridakis M, Christodoulidis G, Chatzitheofilou C, Symeonidis D, Tepetes K (2009). The role of the platelet-rich plasma in accelerating the wound-healing process and recovery in patients being operated for pilonidal sinus disease: preliminary results. World J Surg.

[20] (2022). Scottish Intercollegiate Guidelines Network. SIGN methodology checklist for randomised controlled trials. https://www.sign.ac.uk/what-we-do/methodology/checklists/..

[21] Higgins JPT, Thomas J, Chandler J, Cumpston M, Li T, Page MJ, Welch VA (2019). Cochrane Handbook for Systematic Reviews of Interventions, 2nd Edition.

